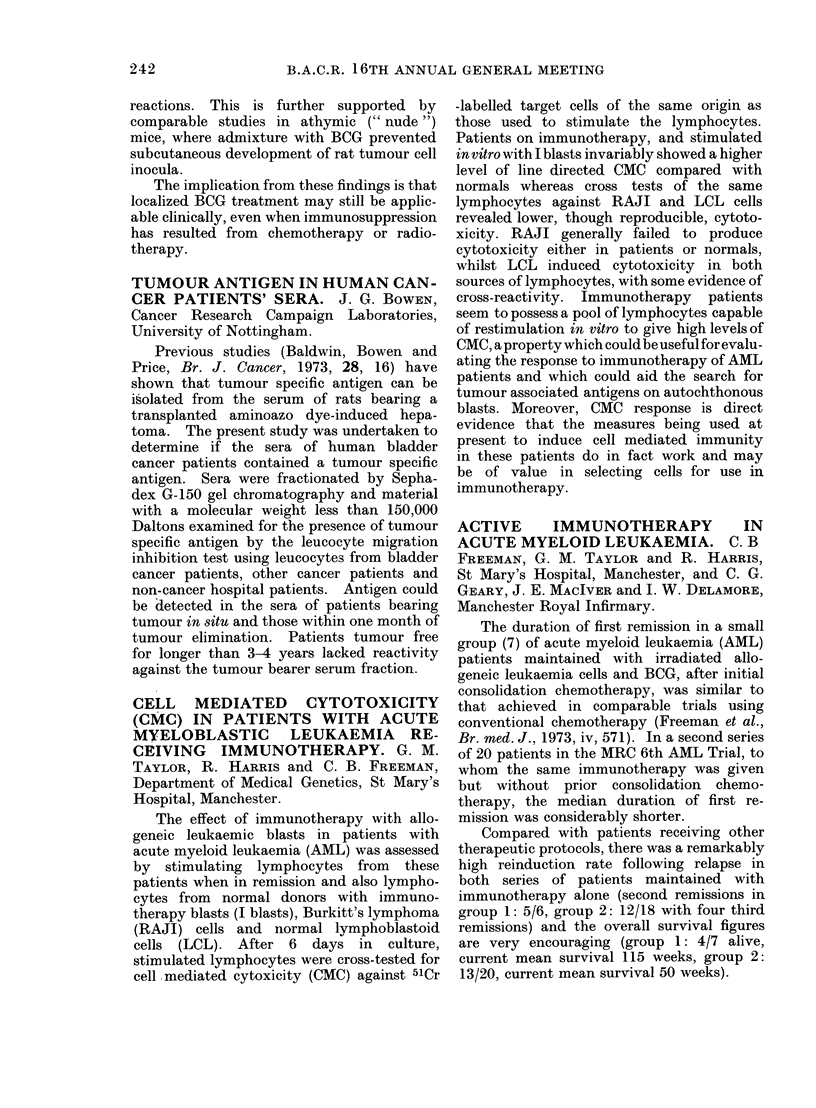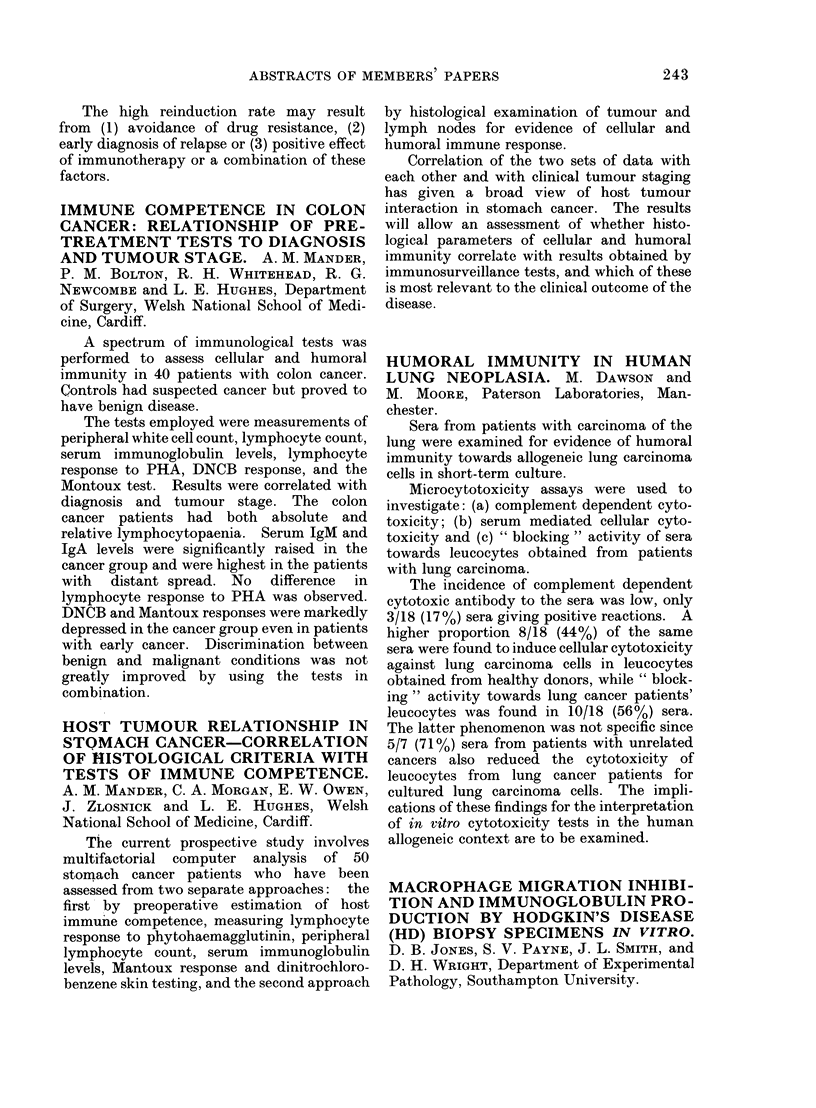# Proceedings: Active immunotherapy in acute myeloid leukaemia.

**DOI:** 10.1038/bjc.1975.163

**Published:** 1975-08

**Authors:** C. B. Freeman, G. M. Taylor, R. Harris, C. G. Geary, J. E. MacIver, I. W. Delamore


**ACTIVE IMMUNOTHERAPY IN ACUTE MYELOID LEUKAEMIA.** C. B Freeman, G. M. Taylor and R. Harris, St Mary's Hospital, Manchester, and C. G. Geary, J. E. MacIver and I. W. Delamore, Manchester Royal Infirmary.

The duration of first remission in a small group (7) of acute myeloid leukaemia (AML) patients maintained with irradiated allogeneic leukaemia cells and BCG, after initial consolidation chemotherapy, was similar to that achieved in comparable trials using conventional chemotherapy (Freeman *et al.*, *Br. med. J.*, 1973, iv, 571). In a second series of 20 patients in the MRC 6th AML Trial, to whom the same immunotherapy was given but without prior consolidation chemotherapy, the median duration of first remission was considerably shorter.

Compared with patients receiving other therapeutic protocols, there was a remarkably high reinduction rate following relapse in both series of patients maintained with immunotherapy alone (second remissions in group 1: 5/6, group 2: 12/18 with four third remissions) and the overall survival figures are very encouraging (group 1: 4/7 alive, current mean survival 115 weeks, group 2: 13/20, current mean survival 50 weeks).

The high reinduction rate may result from (1) avoidance of drug resistance, (2) early diagnosis of relapse or (3) positive effect of immunotherapy or a combination of these factors.